# Help-Seeking as a Maladaptive Coping Style in the Pandemic Scenario: What Worked and What Did Not for Facing This New Stressor

**DOI:** 10.3390/ijerph19010319

**Published:** 2021-12-29

**Authors:** Luca Simione, Camilla Gnagnarella, Giulia Spina, Giuseppe Bersani

**Affiliations:** 1 Istituto di Scienze e Tecnologie della Cognizione, Consiglio Nazionale delle Ricerche, 00185 Rome, Italy; 2 Dipartimento Salute Mentale e Dipendenze, Azienda Socio-sanitaria Territoriale Valtellina e Alto Lario, 23100 Sondrio, Italy; camilla.gnagnarella@asst-val.it; 3 Dipartimento di Scienze Cliniche e Sperimentali, Università degli Studi di Brescia, 25121 Brescia, Italy; spina.giuliapsi@gmail.com; 4 Dipartimento di Scienze e Biotecnologie Medico-chirurgiche, “Sapienza”, Università di Roma, 00185 Rome, Italy; giuseppe.bersani@uniroma1.it

**Keywords:** coping, Brief COPE, stress, COVID-19, lockdown, psychological symptoms, help-seeking

## Abstract

The spread of COVID-19 and its related confinement measures were important stressors for a large part of the global population, with massive effects on both physical and mental health. Assessing how individuals coped with such a stressor and which strategies were effective is one of the main challenges for psychological research. In this study, we aimed to investigate the coping strategies implied during the COVID-19 lockdown and their effectiveness. We recruited 374 Italian participants through convenience sampling during the first pandemic wave (April 2020). We administered to our participants an online battery of questionnaires including the Brief COPE, the use of alternative coping strategies proposed by the WHO to help people facing lockdown stress, and a range of psychological symptoms. An exploratory factor analysis conducted on the subscales of the Brief COPE revealed a three-factor structure. Following the previous literature, we named these factors engagement, disengagement, and help-seeking coping styles. In the pandemic scenario, the engagement and disengagement styles revealed the typical correlation patterns with psychological symptoms (i.e., the engagement was adaptive while the disengagement was maladaptive). Instead, contrary to previous literature, help-seeking was positively related to psychological symptoms, suggesting a mismatch between searching for help and finding it during the lockdown. This result supports the importance of evaluating the effectiveness of coping strategies in the pandemic scenario, to give more compelling and precise advice to the population.

## 1. Introduction

In 2019, the COVID-19 pandemic spread throughout the globe. In particular, it reached Italy as the first country outside Asia, with an impressive number of cases and patients with severe symptoms during March and April 2020 [1]. The national healthcare system also went through a huge crisis due to an increase in the number of hospitalized patients with COVID-19, with difficulties in both containing the virus spread inside hospitals and treating patients with other diseases. In fact, on 11 March 2020, 1028 beds out of the total of 5200 beds in intensive care units were already occupied by patients with COVID-19 [1], with enormous management problems and assistance limitations for patients not affected by COVID-19. In addition to the obvious impact on physical health, the virus had also an impact on mental health. Past outbreaks of a new virus or disease precipitated psychiatric symptoms, worsening psychological conditions, and even led to suicidality [2]. The spread of COVID-19 could be considered a huge stressor as the previous outbreak of new diseases, and it could increase psychological distress and lead to acute and posttraumatic stress disorders (PTSD). The disease outbreak could also have other stressful consequences, such as managing disease symptoms, fear for personal safety and well-being of colleagues and family [3], or experiencing the loss of a loved one during the outbreak [4].

In order to limit the virus spreading, many national governments (included the Italian one) implemented severe restrictions in social and economic life. To contain the virus spread, since 23 February 2020 the Italian Government implemented increasingly restrictive dispositions, up to strong or complete limitations of social life from the middle of March to the end of April. We call the complex of such severe restrictive measures ‘lockdown’. Lockdown had a psychological impact in terms of stress and psychological symptoms. As reported by Brooks et al. [5], previous occurrences of lockdown measures caused negative psychological effects, increasing the risk of acute stress disorder and the later incidence of PTSD, with consequences also in the very long run. Collective stress due to the lockdown could evoke a psychological reaction [5], which could lead to emotional distress in terms of stress [6], anxiety [7,8], and depression [8,9], but also to more notable psychological conditions (even up to psychotic symptoms) [10]. One of the stronger predictors of the stress-induced by quarantine was its duration [11], which could be an important factor also in the COVID-19 scenario due to the very long duration of the lockdown and to the impossibility of defining its conclusion a priori. Therefore, after the initial stress related to the virus outbreak and contagious fear, that related to lockdown and containment measures started to emerge, adding more and more distress to the population that should cope with this new global threat.

### 1.1. Understanding How to Cope with Lockdown-Related Stress Is Fundamental

In an influential position paper, Holmes et al. [12] proposed a call to action to identify “mechanisms (e.g., coping strategies and preventive interventions) to support vulnerable groups under pandemic conditions, such as front-line health and social care staff, those with preexisting mental health issues, young people (aged ≤ 18 years), and older adults (aged 65 years)” [12] (p. 556). In particular, situational coping strategies should be evaluated (that is, coping strategies implied in a specific situation [13] such as the pandemic scenario). Situational coping responses could be affected by many socioeconomic [14] or psychological factors, such as the dispositional coping style [15] or the perceived controllability of a stressor event [16]. Therefore, evaluating the situational coping strategy implied in this completely new scenario due to the global spread of COVID-19 is an open and important issue for psychological research.

One of the most widely used measures of coping strategies is the coping orientation to problem experienced inventory (COPE) [17] and its short form, the Brief-COPE (BC) [18]. Both are multidimensional self-assessing instruments and include a list of possible behaviors or thoughts that people could use for dealing with a stressor. The BC measure 14 coping strategies: self-distraction, active coping, denial, substance use, use of emotional support, use of instrumental support, behavioral disengagement, venting, positive reframing, planning, humor, acceptance, religion, and self-blame. Coping strategies could eventually be grouped into second-order factors or coping styles, each one including multiple coping strategies. Carver et al. [17] proposed the differentiation between an approaching coping style, based on strategies for actively dealing with stressors and their related emotional response, and an avoidant coping style, in which the coping strategies aim to avoid stressors or to not face their emotional consequences. Usually, the former is defined as adaptive and related to a better psychological condition, while the latter is defined as maladaptive and related to a worst psychological condition in a variety of conditions [19]. Based on a factorial analysis on the COPE inventory, Litman [20] added a third factor (i.e., the socially-support coping style), that included the strategies for emotional support, instrumental social support, and venting emotions. This style focused on searching social support and it was reported as adaptive (i.e., related to increased psychological wellbeing). The same factorial structure was found by Gutierrez et al. [21] for the BC, and they named the three coping styles engagement, disengagement, and help-seeking.

In the pandemic scenario, previous studies have already shown that the two main coping styles (i.e., approaching/engagement and avoidant/disengagement) had the expected role, respectively, in reducing and increasing psychological distress [22,23,24]. However, the role of the socially-supported/help-seeking style was not investigated. This is particularly needed considering the impact of COVID-19 and quarantine measures on social isolation, and how this could, in turn, impact mental health. Previous studies related to the COVID-19 pandemic reported that perceived social support was negatively related to depression [25], psychological distress [26], and anxiety [27]. Fugui et al. [28] suggested that social support would buffer the negative impact of low resilience on mental health, again with a positive effect on psychological conditions. However, Saltzman et al. [29] argued that, in times of social isolation, coping based on social support requires that others change their way of participating in social life, such as using social networks. Thus, searching for social support could not equate to finding such support. In this regard, Zhi et al. [30] showed how pandemic-related experiences reduced perceived social support, thus increasing acute stress symptoms. In this complex scenario, in which pandemic and lockdown affected the social life and changed how people interacted with each other, it is important to assess the effect of socially-oriented coping on mental health. In fact, this coping style could fail if social support was needed but not found.

### 1.2. Alternative Strategies Suggested by Public Health Institutes

The BC investigates a range of possible coping strategies, but not all of them. More specifically, the WHO [31] proposed several strategies to help people facing stress related to the COVID-19 pandemic. The WHO’s advice included spending time and remaining in contact with family members or friends (also by email or phone), thus encouraging the maintenance of proper social support as an important resilience factor [32]. Moreover, it suggested individuals maintain a healthy lifestyle and dedicate themselves to hobbies or pleasant activities, avoid smoking, alcohol, or drugs, take information only from credited sources, and use the same skills or strategies that have already worked in past for managing emotions during tough times. The Ministry of Health in Italy [33] also relaunched this advice, encouraging people to keep in touch with familiars and to have a healthy lifestyle. These strategies were presented by credited sources and therefore adopted by many people, but their effectiveness in managing COVID-19-related stress should be verified to evaluate their use in similar future scenarios.

### 1.3. The Present Research

Understanding all of these factors appears to be crucial to effectively managing the psychological conditions associated with COVID-19-related stress [12], therefore, we designed and conducted a cross-sectional study involving an Italian sample from the general population during the first phase of the pandemic spread. We aimed to assess how coping strategies combined in high-order coping styles and which coping styles were effective or ineffective for managing stress during COVID-19 spreading and lockdown limitations. Hanfsting et al. [34] showed that previously proposed models of the BC high-order factorial structure did not fit well data obtained immediately after the end of the national lockdown in Germany and Austria. This result points to the need for a new assessment of the factor structure of BC during the pandemic.

To this aim, we assessed the coping strategies used by participants through the BC and a customized questionnaire investigating the strategies proposed by institutional actors at the beginning of the pandemic. We further investigated whether and how coping strategies cluster together in higher-order coping styles and if they were adaptive or maladaptive in such a scenario. Then we assessed their effect on both psychological stress and a range of psychological symptoms that proved to be influenced after the spread of COVID-19, such as anxiety [7,8], depression, death and disease anxiety [35], somatization [36], anger [37], paranoid ideation [38], and psychotic symptoms [10].

Based on the reviewed literature, our first hypothesis was that our sample would report a moderate to high level of stress and distress as a consequence of pandemic spread and lockdown. About BC, we hypothesized that the coping scales would load onto two or three factors [21,39]. We hypothesized that an approach or engaging coping style would be related to reduced psychological symptoms and distress, while an avoidant or disengaging coping style would be related to increased psychological distress. In a three-factor model, a socially-oriented or help-seeking coping style could have an adaptive effect (i.e., it would negatively correlate with the presence of psychological symptoms). However, it could be also possible that socially-oriented coping could be positively related to psychological symptoms, indicating a mismatch between seeking support and finding it [29]. Finally, we would expect that the use of alternative strategies suggested by public health authorities would be related to decreased psychological distress.

## 2. Materials and Methods

### 2.1. Participants

We enrolled 374 adult participants, voluntarily recruited during April 2020 with an online snowball sampling using email and social media. There was no remuneration for participation. From the original sample, we excluded 18 univariate outliers (see Results for details), obtaining a final sample of 356 participants (mean age = 40.97 years, SD = 11.04 years, females = 298, males = 58) for the analysis. In this sample, 106 participants were in remote working, while the remaining worked as usual. 29 participants reported the presence of at least one psychic condition, such as anxiety disorders, panic attacks, and mood disorders. A total of 71 participants reported at least one medical condition associated with an increased risk in the case of COVID-19 (mean = 0.27).

Regarding their experience with COVID-19, eight participants were infected (they reported being positive for a COVID-19 swab), one has been hospitalized following a COVID-19 infection, 78 reported that one of their familiars or close friends contracted COVID-19, and 19 that a familiar or close friend died from COVID-19.

### 2.2. Procedure

Participants read an information note about the study and provided their informed consent. Then, they compiled a battery of questionnaires as described in the next section. Each questionnaire was presented in an online module, and the next one appeared after completing the previous. In the first module, demographic information was collected. We then asked participants to report their activities during the lockdown and their housing conditions. Afterward, they complete the psychological questionnaires (see Section 2.3). All data were collected in a completely anonymous format.

This study was performed in accordance with the Declaration of Helsinki. This human study was approved by the Research Ethics and Integrity Committee of the CNR. All adult participants provided informed consent to participate in this study.

### 2.3. Measures

First, we collected demographic info, including age, sex, and level of education. We asked for the working status (i.e., if working as usual or if in remote working), coded as 0 = working as usual and 1 = in remote working. We asked for the presence of psychic conditions, coded as 0 = no psychic condition and 1 = presence of one or more psychic condition. We also checked for the presence of medical conditions associated with an increased risk in the case of COVID-19 using a checklist including cardiovascular diseases, diabetes mellitus, hypertension, chronic pneumopathies, neoplasms, immunodeficiencies, hematological pathologies, and neuromuscular diseases. We computed a medical status index by summing all of the checked items, ranging from zero to eight. Participants also reported their living conditions during the lockdown by checking all of the home issues that applied to their situation from the following list: insufficient square meters for the number of residents, lack of a private space/privacy, lack of outdoor spaces, excessive humidity, excessive noise, excessive pollution, floor too low/basement, noisy or annoying neighbors, conflict within the home, poor essential services, excessive isolation, presence of people who require special assistance, lack of internet. We computed a house distress index (HDI) by summing all of the checked items, with a score ranging from 0 to 13. Participants should also report on a four-point Likert scale (from ‘never’ to ‘everyday’) how much time they spent time doing different activities (i.e., physical training outdoor, physical training at home, hobbies practice, relaxation or meditation, enjoying their family). We also collected information about personal experience with COVID-19. In particular, participants reported if they contracted COVID-19, if they were hospitalized due to COVID-19 infection, if a familiar or close friend contracted COVID-19, and if a familiar or close friend died due to COVID-19.

Then, we tested participants through ten questionnaires. We measured coping strategies through BC [18], a 28-item questionnaire that included 14 two-item subscales. An Italian situational version of the BC was developed by Monzani et al. [40]. They reported how the 14 strategies were highly correlated with each other, suggesting that some form of second-order grouping could exist between such strategies. In BC, each scale evaluates the use of a particular coping strategy: self-distraction, active coping, denial, substance use, use of emotional support, use of instrumental support, behavioral disengagement, venting, positive reframing, planning, humor, acceptance, religion, and self-blame. Cronbach’s alpha in our sample for these scales was in the range of 0.33 (self-blame scale) to 0.91 (substance use scale). Poor Cronbach’s alpha was revealed for self-distraction, self-blame, humor, behavioral disengagement, and venting. However, the reliability of two-item scales usually did not accurately reflect the true reliability [41].

We then measured psychological distress through four scales: the Perceived Stress Scale (PSS) [42], a 10-item scale measuring how much participants perceive their lives as unpredictable or out of control; the short form of State-Trait Anxiety Inventory (STAI) [43], a six-item questionnaire exploring anxiety and distress; the General Health Questionnaire (GHQ) [44], 12-item version, a questionnaire investigating depressive and distress symptoms; the ‘death anxiety’ scale of the Existential Concern Questionnaire (ECQ) [45], a five-item scale exploring the anxiety due to death, illness, and unpredictability of life. All of these four scales showed good to excellent reliability, with Cronbach’s alpha ranging from 0.81 to 0.90.

Furthermore, we also evaluated psychological symptoms using four scales from the Symptoms Checklist 90-R (SCL-90R) [46]. In particular, we administered scales measuring hostility/anger (6 items), paranoid ideation (6 items), psychoticism (10 items), and somatization (12 items). These scales showed good reliability, with Cronbach’s alpha ranging from 0.81 to 0.91.

Lastly, we also administered the Holmes-Rahe stress inventory (HR) [47], a checklist of 43 possible stressful events that evaluated the total stress level of participants as the weighted sum of the events reported. We measured the events that happened in the last month, i.e., from the beginning of the lockdown.

### 2.4. Data Analysis

For each scale, we computed descriptive statistics. Then we checked the presence of univariate outliers for each scale (data point exceeding ± 2 SD from the mean). We managed outliers with case-wise deletion.

For BC, we conducted a series of exploratory factor analyses (EFA) at the scale level of the BC to assess the structure of coping styles in our sample. We used the extraction methods of principal components and we applied two rotation methods i.e., *varimax* (assuming independent factors) and *oblimin* (assuming related factors). We first assessed the number of dimensions suggested to be extracted with scree analysis and BIC testing solutions that included 1 to 10 factors. After this step, we selected the best solution and analyzed its factor structure, considering adequate loadings > 0.40 and uniqueness < 0.60. This further check allowed for the selection of the final model to be implied in the successive analysis. If we did not find any satisfying solution, we would use the basic fourteen scales. We would present descriptive statistics and plots to assess the coping style patterns adopted by participants in our study.

Afterwards, we assessed the association between coping and mental health by mean of bivariate Pearson correlations. We also assessed the relationship pattern between mental health scores and other measures, such as demographic variables and alternative coping strategies not evaluated with the BC. As we conducted a great number of correlations in a relatively large sample, we considered a correlation coefficient as meaningful if it was significant at level α = 0.01 and if the coefficient was greater than 0.20 or less than −0.20. In addition, we assessed the pattern of relationships between coping and symptoms with semi-partial correlation analysis controlling for the effect of covariates (that is, demographic variables, lockdown activities, and stress factors). In this manner, we identified the effective relationship of the different coping styles to participants’ mental health.

We also analyzed the effect of personal experience with COVID-19 on coping styles. Thus, we conducted a series of independent sample t-tests in which we compared on coping styles participants who contracted COVID-19 with participants who did not, and participants who had a family member or a close friend who contracted or died from COVID-19 with participants who did not report these experiences. We did not analyze the effect of being hospitalized due to COVID-19, as only one participant reported this experience.

All of the analyses reported in this paper were performed in R version 3.6.3 (R Foundation for Statistical Computing, Vienna, Austria).

## 3. Results

### 3.1. Descriptive Statistics

From the original sample, we identified and removed 18 univariate outliers. Table 1 reports descriptive statistics for demographics and lockdown activity variables calculated after removing univariate outliers. As shown, most of the variables reported an acceptable normal distribution. However, some BC and SCL-90R scales showed positive and high values for skewness and kurtosis. Such high values suggested a left-skewed distribution. In particular, this was true for the substance use subscale, for which participants reported mostly a zero score. To overcome such non-normality, we implied a robust model estimator for the EFA. Instead, bivariate correlations are not affected by nonnormal data.

About the psychological condition of our participants, we noticed that the average PSS score was in the range of mild stress, as > 13. Moreover, also average general distress score, as measured by GHQ-12, was higher than the threshold score of 11–12 usually implied to detect psychiatric cases, in particular for PTSD [48]. Then, participants reported on average a consistent level of distress during the first wave of pandemic spread.

### 3.2. Brief-COPE Factor Structure

We analyzed the higher-order factor structure of the BC. To this aim, we conducted a factor analysis on the first-order scales. Bartlett’s test of sphericity χ^2^ (91) = 1214.32, *p* < 0.01, indicated that the correlation structure was adequate for factor analyses. Both the scree-test and the BIC suggested the presence of three factors. We then conducted an EFA exploring a three-factor solution with oblimin rotation. The result of this analysis is reported in Table 2.

As reported, we obtained a three-factor interpretable solution with high loadings (>0.40) and low uniqueness (<0.60) items. The solution explained 49% of the variance. In particular, we found an engagement (or approaching) coping style, explaining 20% of the variance and including five scales (i.e., acceptance, positive reframing, planning, humor, and active coping). The second factor explaining 18% of the variance was the help-seeking coping style, including three scales (i.e., use of instrumental support, use of emotional support, and venting). Lastly, a third coping style was identified as disengagement (or avoidant) coping, which explained 11% of the variance and included behavioral disengagement and denial. The items self-distraction, self-blame, religion, and substance use were discarded from the three factors as they both showed low factor loadings and high uniqueness scoring. An additional analysis after the removal of the high-uniqueness items produced a further three-factor solution explaining 62% of the variance without changes in the structure. The use of the varimax rotation (i.e., implying non-correlated factors) did not modify the results substantially.

For the successive analyses, we implied the three second-order coping styles that emerged in this factor analysis (i.e., engagement, help-seeking, and disengagement). We further analyzed the three coping styles by inspecting their reciprocal correlation and their correlation patterns with psychological outcomes. Table 3 reports the result of this correlation analysis. Engagement and disengagement were weakly and negatively correlated, r = −0.16, whereas help-seeking was positively correlated with both engagement, r = 0.29, and disengagement, r = 0.21. In terms of psychological symptoms, engagement coping was related to better psychological conditions. The use of engagement coping styles was few or not related to the symptomatologic dimensions measured with the SCL-90R. In contrast, disengagement coping was strongly related to increased psychological symptoms in all of the investigated areas. The third coping style, help-seeking, was also associated with an increased level of psychological symptoms. Although both disengagement and help-seeking were related to increased psychological distress, the former was related to the worst psychological conditions, as indicated by higher correlation coefficients.

**Figure 1:**
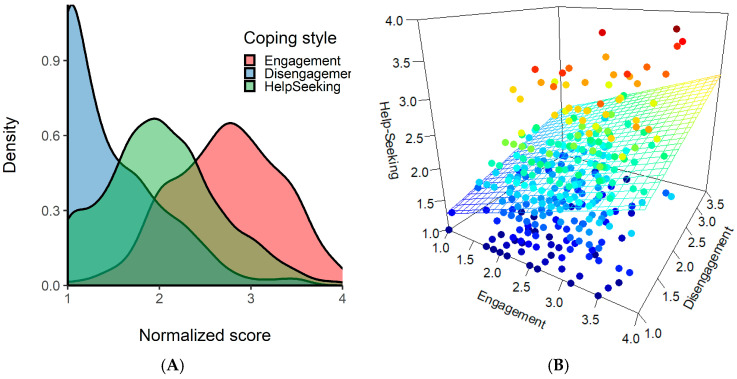
Panel (**A**): density plot of the scores for the three coping styles in our sample. The scores were normalized in the range from 1 to 4. Panel (**B**): 3D scatterplot of the relationship between engagement (*x*-axis), disengagement (*y*-axis), and help-seeking (*z*-axis) coping style. Each point represents a participant and the color of the points maps the help-seeking score, from blue (lower score) to red (higher score). The regression surface represents the relationship of help-seeking with engagement and disengagement styles [49].

We then analyzed the distribution of the three coping strategies in our sample and how they combine in each participant. To this aim, we computed the distribution of the coping styles scores in the sample. Figure 1A depicted the density plot relatively to the three coping styles, showing that engagement style was distributed around higher values, help-seeking style showed intermediate scores, and disengagement style showed a distribution left-skewed toward the minimum. This analysis showed how participants used mostly engagement strategies while they tended to use less a disengagement style. Help-seeking was also a strategy that our participants largely implied. To study the relationship between the coping styles, we produced a 3D scatterplot (Figure 1) in which we reported the combination of the three coping styles reported by each participant. As shown, participants tended to use either engagement or disengagement coping as the main style, and then they could combine it with the help-seeking style or not. In fact, participants tended not to report using only help-seeking without another style. Some participants reported using none or all of the coping styles. This analysis revealed the role of help-seeking as secondary more than as principal coping style.

### 3.3. Relationships between Coping, Socio-Demographic, and Psychological Variables

After assessing the factor structure of the coping styles in our sample, we performed bivariate Pearson correlations between coping styles, socio-demographic, and psychological variables to assess their relationships. In Table 4 we presented the correlation pattern between demographic variables, lockdown activity, and stress factors with coping styles and psychological outcomes. As reported, education level was related to decreased use of disengagement coping style. About the psychological variables, the presence of a psychic condition, stressful housing condition, and the presence of stressful events were associated with increased psychological symptoms. Instead, age, education level, and spending time on hobbies were associated with decreased psychological symptoms. Lastly, the occurrence of stressful events and HDI were strongly associated with the presence of a range of psychological symptoms, but not with the use of particular coping styles. These significant correlations between stress factors and psychological variables suggested the importance of controlling their effects while evaluating the association between coping styles and mental health, as we reported afterward.

Then we assessed the effect of the COVID-19 variables on the coping styles. First, we compared participants who had COVID-19 (*n* = 8) with participants who did not (*n* = 348). The two groups did not differ in the use of any coping style, *p* > 0.20 for all contrasts. We then compared participants with a familiar or close friend who contracted COVID-19 (*n* = 78) with the others (*n* = 278). Again, we found no difference between the two groups for any coping style, *p* > 0.27 for all contrast. Lastly, we compared participants who lost a familiar or close friend for COVID-19 (*n* = 19) with the others (*n* = 337), and again we did not find significant differences in the use of coping style, *p* > 0.27 for all contrasts.

We also computed semi-partial correlations between the three coping styles and the psychological outcomes, controlling for demographic variables, lockdown activities, and stress factors, i.e., HDI and HR-stress index. The coefficients for these analyses were reported in Table 5. As reported, the coping styles were strongly related to the considered psychological variables. In particular, relying on an engaging coping style was related to a reduction in stress, anxiety, and general distress, while relying on a disengagement or help-seeking coping style was related to increased symptoms in all of the investigated dimensions.

### 3.4. Control Analysis on Help-Seeking: The Role of Venting

Our results showed a positive correlation between help-seeking and psychological symptoms. As this coping style was expected to relate to a better psychological condition [20], we performed a control analysis to assess whether the inclusion of the venting strategy in help-seeking style determined this positive correlation. While venting was usually included in this style [20,21,39], it was also categorized as a maladaptive coping strategy [50]. Thus, we conducted again all of the analyses presented so far with a different help-seeking score, in which we included only the strategies use of emotional support and use of instrumental support. We found that all of the main patterns of results held equally with the help-seeking score without venting. This new score was strongly positively correlated with the old one, r = 0.95, *p* < 0.01, and it was positively correlated with both engagement, r = 0.25, *p* < 0.01, and disengagement style, r = 0.20, *p* < 0.01. Moreover, it showed the same pattern of correlations with mental health variables, with all significant positive relationships, rs, ranging from 0.14 to 0.27.

## 4. Discussion

In this paper, we investigated how people coped with the distress due to COVID-19 spreading and to lockdown. To this aim, we administered the BC to a sample of Italian participants together with other questionnaires assessing a range of psychological symptoms, including stress, anxiety, death anxiety, depression, somatization, anger, psychoticism, and paranoia. We evaluated the factor structure of the coping scale and then correlated the resulting coping styles with the demographic and psychological outcome measures.

In accordance with previous studies [21,39], we found three main coping styles. Following Gutiérrez et al. [21], we named these three coping styles engagement, disengagement, and help-seeking. These factors have already been reported in the literature with different names, for example self-sufficient, avoidant-coping, and socially-supported coping strategies [20]. Unlike similar studies investigating the relationship between COVID-19-related stress and coping strategies, our factor analysis allowed us to assess how coping strategies were combined during the pandemic instead of applying a preexisting high-order model of coping styles. Previous studies on this topic [23] implied the classic 2-factor structure in which coping strategies were classified as adaptive or maladaptive [17]. However, this approach had two main flaws. First, from a theoretical point of view, it over-simplified the relative complexity of the possible combination of approaches to coping with a stressor [51]. This criticism could also be applied to other studies on COVID-19 in which coping strategies were grouped as approaching and avoidant strategies [22,24]. Second, from a practical point of view, the pandemic could be considered a completely new stressor [52]. Therefore xsdc xd x, it could be hazardous to consider some strategies a priori as effective or ineffective in such a new scenario.

### 4.1. Help-Seeking as a Maladaptive Coping Style during the Lockdown

In line with this point, we found an unexpected result about the effectiveness of coping styles. While engagement and disengagement coping style showed their typical pattern of results [17], help-seeking coping style was related to higher levels of psychological symptoms. We also found that help-seeking style was rarely used as the main coping style, but it was used mainly in combination with either engagement or disengagement coping styles. We should expect that socially-oriented strategies were related to better psychological conditions [53], or that they were not related to increased psychological symptoms [54], whereas a positive association with them was not expected. In line with this expectation, the WHO suggested contacting family members or friends during lockdown to cope with COVID-related stress [31]. Instead, our result pointed out how, during the first Italian lockdown, individuals who tend to cope by seeking social support from others reported negative psychological conditions as those using disengagement or avoidant coping. Interestingly, this result was true also among individuals using an engagement style. However, it is possible that people with worse mental health conditions used help-seeking strategies more than people with better psychological condition [55]. Following this possible explanation, using help-seeking strategies could not be considered as the cause of worst psychological conditions, but better as a co-occurred phenomenon or even a consequence. In fact, distressed people could seek social support to ease their problems. As our study was limited by its cross-sectional design, we could not disentangle this causality pattern and thus we should interpret our results with caution. We propose three possible explanations for this pattern of results below.

First, social support seeking as a style can be affected by stressor characteristics [54], therefore, the specific characteristics of lockdown as a stressor should be considered. In fact, during the lockdown period, most social life events were not allowed or limited, and many people were left alone or isolated. In this condition, social contacts were difficult. While contacting friends by phone or the Internet was possible, our result seems to suggest that online contacts could not have the same effectiveness as the ones in presence and that they could also exacerbate loneliness and distress [56]. It is possible that studies conducted in other period of the pandemic would find a different pattern of results. In fact, a study with a very large cross-regional sample collected from April to June 2020 [57] found that social support strategies positively mediated the effect of perceived severity of COVID-19 disease and mental health, with perception of illness that increased social support seeking, which in turn increased well-being. This result was in contradiction with ours, but our data was collected in April 2020 while their data [57] was collected later, when most of the restrictive measures relaxed over time in Italy as in many other countries. Thus, it is possible that our result applies to the very first phase of the pandemic, when the most severe social limitations were implied, but not to later ones.

Another possible explanation was that help-seeking was maladaptive since individuals sought social support only to vent their emotional distress [13]. Venting is considered a way to cope with emotions, mainly associated with the expression of negative emotions and increased psychological distress [58]. Also in the COVID-19 scenario, venting was already identified as an ineffective coping strategy and associated with a higher level of stress [59]. However, our control analysis showed that removing venting from the help-seeking score did not alter its pattern of relationships with other variables. Thus, emotional or instrumental support also seemed to be related to increased psychological problems, suggesting a more general maladaptive role for all of the strategies relying on finding out help during the pandemic.

Thus, a third possible explanation could be that seeking social help in this scenario would not be equal to receive social support. The pandemic spread and its related containment measures were stressful, with a consistent increasing of psychic symptoms such as depression, anxiety, and stress among the population. In such a challenging scenario, it could be possible that people showed difficulties in emotionally supporting each other, especially at the very first phase of pandemic spread, characterized by great uncertainty about the future and concrete worries about personal survival and health [60]. While help-seeking was correlated with increased psychological symptoms, measures of perceived social support (i.e., the perception of being effectively supported by others) were positively correlated to psychological well-being and mental health [61]. Thus, a high level of social support perception could be associated with a better psychological condition, showing that this could be considered a protective factor if properly exploited [62]. Based on our results and previous literature, we could further hypothesize that perceived social support and socially-oriented coping style could interact in affecting mental health. Searching for help as a coping strategy would be effective only in case of high perceived social support (i.e., if the social support was effectively provided). Otherwise it could lead to worse psychological conditions than other coping styles, if associated to low perceived social support. Therefore, future studies could address this hypothesis using a measure of perceived social support in combination with BC.

To conclude, we would underline that our results should not be interpreted against the use of help-seeking strategies, but maybe for their better implementation. Increasing social connections and support could be an important resource to face social isolation and loneliness [63], and thus to increase mental health. As also stated by both the European Psychiatric Association [64] and the World Psychiatric Association [63], creating and sustaining social connections should be considered a priority for policy makers to contrast mid- and long-term mental effects of the pandemic. Future research should also investigate the correct way to exploit the new social channels (e.g., social media and online meetings), in order to give people the opportunity to reduce the social gap even during quarantine or isolation and to give healthcare workers new instruments to deliver support when face to face interactions are limited or difficult.

### 4.2. Pandemic and Lockdown Were Faced as Traumatic Events

We found on average a mild to high level of psychological distress in our sample, in line with previous studies carried out during the first pandemic wave in Italy [35] and other countries [65,66]. Compared to previous studies assessing the coping strategies used during the COVID-19 pandemic with the BC, we originally implied a factor analysis to reveal the high order structure of the coping strategies [24,59]. Our analysis revealed that a three-factor model fitted the data well. This model seems to be robust in different types of traumatic events [39]. However, the effectiveness of the three coping styles to face each particular traumatic event could change. Wang et al. [39] found no positive nor negative effect of help-seeking strategies in flood survivors and breast cancer patients, while we found that such coping style was related to increased psychological symptoms. In addition, we found an effect of the engagement style only on some psychological variables, while Wang et al. [39] found a stable and consistent positive effect of the same style in their analysis. Taken together, these results highlight the importance of evaluating both the structure of high-order factors and the adaptiveness of each individual factor based on the particular characteristics of the stressors or traumatic events considered, especially for the situational version of BC [40].

### 4.3. Effectiveness of Alternative Coping Strategies and Activities

In addition, we analyze the association of mental health with alternative coping strategies not included in the BC inventory. We found that training either at home or outdoor was not associated with any psychological outcomes. While physical training has been usually related to enhanced stress responsivity and better mood state [67], it seems not to have worked during the lockdown. Individuals already used to train may have had to change their exercise routines, with a possible increase in the level of stress, while individuals who have not trained before should not have benefited in the short term, since physical training seems to have more a protective effect on stress than a corrective one [67]. Also, spending time in relaxing or funny activities, or taking care of family members seemed not correlated to better psychological conditions. All of these activities could have a positive effect on psychological health, as suggested by WHO [31], and our study could have failed in finding a significant effect due to its methodological limitations (see section below). In line with this, we consistently found negative correlations between these activities and all of the psychological symptoms, but no one was significant. A part for study limitations, this result again corroborated the idea that this scenario was tough to face for the general population and that strategies useful in other situations seemed to be less effective in this new one.

### 4.4. Effect of Housing Condition and Stressful Events

Lastly, we discussed the effect of house-related stress and the occurrence of other stressful events during the lockdown on coping styles and psychological well-being. Interestingly, stress related to the home was positively related to most of the psychological symptoms investigated but did not show any relationship with coping. This result is consistent with previous literature, showing that housing conditions could be related to psychological well-being [68], but it also originally showed that distress related to housing condition did not affect the preference for a set of coping strategies over the others. The occurrence of stressful events during lockdown also showed a positive correlation with psychological symptoms, but no correlation with coping styles. This pattern of results suggests that participants have been coped with stressors that occurred in the new scenario with the same strategies they have used before the pandemic onset. In this sense, this could be a sign of low coping flexibility and could contribute to explaining the struggle reported by people facing pandemic-related stress [69].

This explanation was further supported by the analysis of the effect of direct experiences with COVID-19 on coping styles. We found no difference in coping styles between participants infected and those non-infected, as well as between participants with familiars or close friends who were infected or died of COVID-19 and those without these experiences. This again supported the relative difficulty of participants in adapting their coping style, probably due to participants’ difficulty in correctly evaluating the environment and the situation so as to effectively adapt their coping style to the new stressors [70].

### 4.5. Limitations of Our Study

The main limitation of our study was the cross-sectional design, in which it is not possible to completely unravel the causal relationship pattern between variables. For example, it is possible that people with the worst mental health condition used help-seeking strategies [55], while we hypothesized a path from coping styles to mental health. However, we considered this analysis interesting in any case, as it was grounded in literature and allowed us to uncover the relationship pattern between coping style, psychological, and socio-demographic variables. A second main limitation was the method of using the online convenience sample implied to contact participants. This was a limitation for two main reasons. First, we had no control over the demographic characteristics of the sample. Second, all participants were volunteers and therefore motivated to participate in the research. However, at that time we experienced great limitations in both time (to have data referring to the same context) and space (as laboratories and universities were closed). Thus, we were forced to conduct the study online.

## 5. Conclusions

In this study, we showed that a three-factor high-order structure of coping styles could be considered stable across different stressful events, including the pandemic spread. Moreover, we also confirmed that adaptive and maladaptive strategies could be defined only by considering the complex contextual scenario in which such strategies are implied [39]. In particular, we found how people using a help-seeking style revealed a negative psychological condition regarding a wide range of symptoms. This result could guide future public interventions and advice to the general population. For example, simply encouraging people to stay in contact with their relatives and friends during a quarantine could increase their tendency to focus on negative emotions and venting, with an increase in psychological distress [58]. More compelling advice could help people in searching and offering social support in the right ways, as perceiving social support from others could be an important factor of resilience [62]. Coping with the pandemic spread and lockdown measures is turning out to be stressful for people all around the world [12]. Giving to them the best possible support is crucial to avoid adopting maladaptive coping strategies [71] that could lead to an increased level of psychological suffering worldwide, with even worst consequences on the long run.

## Figures and Tables

**Table 1:** Descriptive statistics for demographic, stress factors, lockdown activity, coping strategy, and psychological symptoms.

	Variable	Mean	SD	Skewness	Kurtosis	Cronbach’α	Outliers
Demographics and others	Age	40.99	11.04	-	-	-	-
	Education (in years)	15.08	4.09	-	-	-	-
	Physical condition	0.27	0.61	-	-	-	-
Stress factors	HDI (house distress index)	1.38	1.53	-	-	-	-
	HR stressful events	113.9	73.13	-	-	-	4
Time spent in…	Training outdoor	0.30	0.68	-	-	-	-
	Training at home	0.98	0.98	-	-	-	-
	Hobby and fun	1.29	0.96	-	-	-	-
	Relaxing activity	0.45	0.81	-	-	-	-
	Taking care of family	1.91	1.12	-	-	-	-
Brief COPE	Self-distraction	5.14	1.57	−0.08	−0.63	0.48	-
	Active coping	5.17	1.56	−0.07	−0.66	0.63	-
	Denial	3.02	1.46	1.54	1.75	0.78	-
	Substance use	2.18	0.71	5.13	30.86	0.91	-
	Use of emotional support	3.97	1.55	0.56	−0.21	0.86	-
	Use of instrumental support	3.98	1.47	0.55	−0.01	0.72	-
	Behavioral disengagement	2.89	1.15	1.31	1.44	0.54	-
	Venting	4.23	1.31	0.14	−0.38	0.49	-
	Positive reframing	5.13	1.60	0.01	−0.72	0.76	-
	Planning	5.31	1.61	−0.13	−0.70	0.71	-
	Humor	3.61	1.34	0.71	0.01	0.56	-
	Acceptance	6.29	1.49	−0.55	−0.38	0.80	-
	Religion	3.41	1.68	1.00	0.03	0.84	-
	Self-blame	3.74	1.13	0.77	1.06	0.33	-
Psychological symptoms	PSS	19.33	6.63	0.14	−0.26	0.81	-
	STAI	14.27	4.24	0.03	−0.78	0.87	-
	ECQ Death anxiety	8.63	5.70	0.35	−0.91	0.90	-
	GHQ	16.77	4.86	−0.23	−0.10	0.86	-
	SCL-90R Som	14.06	10.46	0.79	−0.09	0.91	1
	SCL-90R Hos	4.48	4.05	1.23	1.08	0.85	3
	SCL-90R Psy	5.38	5.66	1.31	1.06	0.81	6
	SCL-90R Par	4.90	4.58	1.02	0.34	0.84	4

Note. SD, standard deviation; HR, Holmes-Rae stress index; PSS, perceived stress scale; STAI, state-trait anxiety inventory; ECQ, existential concerns questionnaire; GHQ, general health questionnaire; Som, somatization; Hos, hostility/anger; Psy, psychoticism; Par, paranoia. The total sample after outlier removal includes 354 participants, with 296 females and 58 males. The skewness and kurtosis values underlined indicate a suspect toward nonnormality.

**Table 2:** High-order factor structure of the Brief COPE scales.

	Factor 1	Factor 2	Factor 3	
Brief COPE Scale	Engagement	Help-Seeking	Disengagement	Uniqueness
Acceptance	**0.70**	−0.14	−0.12	0.50
Positive reframing	**0.70**	0.10	−0.09	0.48
Planning	**0.69**	0.25	−0.09	0.41
Humor	**0.65**	−0.31	0.38	0.42
Active coping	**0.60**	0.27	−0.21	0.49
Use of instrumental support	0.05	**0.86**	0.03	0.24
Use of emotional support	−0.04	**0.86**	0.01	0.28
Venting	0.17	**0.61**	0.15	0.53
Behavioral disengagement	−0.04	0.01	**0.84**	0.29
Denial	−0.26	0.31	**0.58**	0.49
Self-distraction	0.37	0.28	0.33	**0.63**
Self-blame	0.40	0.25	0.31	**0.64**
Religion	0.23	0.34	−0.08	**0.81**
Substance use	0.10	−0.14	0.22	**0.93**
Cumulative variance	0.20	0.38	0.49	
Proportion explained	0.37	0.40	0.23	

Note. Loadings above 0.40 are marked in bold. Uniqueness above 0.60 is in boldface.

**Table 3:** Correlation analysis of the three identified coping styles.

	Engagement	Disengagement	Help-Seeking
Engagement	-	−0.16	**0.29**
Disengagement	-	-	**0.21**
PSS	**−0.20**	**0.38**	**0.28**
STAI	**−0.25**	**0.36**	**0.31**
ECQ Death anxiety	−0.06	**0.34**	**0.27**
GHQ	**−0.22**	**0.29**	0.16
SCL-90R Som	−0.05	**0.29**	**0.28**
SCL-90R Hos	−0.10	**0.20**	**0.20**
SCL-90R Psy	−0.13	**0.41**	**0.27**
SCL-90R Par	−0.06	**0.24**	0.18

Note. PSS, perceived stress scale; STAI, state-trait anxiety inventory; ECQ, existential concerns questionnaire; GHQ, general health questionnaire; Som, somatization; Hos, hostility/anger; Psy, psychoticism; Par, paranoia. Coefficients marked in boldface are *p* > 0.20 or < −0.20, and significant at *p* < 0.01.

**Table 4:** Bivariate correlation coefficients between demographic variables, lockdown activities, stress factors, and coping styles.

	Demographic Variables						Lockdown Activities					Stress Factors	
	Sex	Age	Edu.	RW	Psychic Condition	Physical Condition	Training Outdoor	Training at Home	Hobby and Fun	Relax	Family	HDI	HR
Engagement	−0.01	0.06	0.17	0.09	−0.11	0.12	0.05	0.12	0.16	0.14	0.15	−0.01	0.10
Disengagement	0.07	0.09	**−0.20**	−0.10	0.17	0.11	0.02	−0.08	−0.19	−0.01	−0.08	0.05	0.17
Help-Seeking	0.16	−0.13	0.02	−0.04	0.03	−0.02	−0.09	0.05	−0.04	0.03	0.03	0.10	0.19
PSS	0.14	−0.19	−0.18	−0.10	**0.22**	0.01	−0.04	−0.11	−0.14	−0.15	−0.12	**0.29**	**0.28**
STAI	0.12	−0.16	−0.12	−0.09	**0.23**	0.03	−0.07	−0.09	−0.16	−0.13	−0.13	**0.30**	**0.28**
ECQ Death anx.	0.14	0.01	−0.17	−0.07	0.16	0.09	−0.06	−0.11	−0.09	−0.13	−0.06	0.17	0.19
GHQ	0.03	−0.10	−0.10	−0.11	0.07	0.01	−0.11	−0.11	**−0.20**	−0.19	−0.14	0.18	0.05
SCL-90R Som	0.19	−0.13	**−0.24**	−0.08	**0.21**	0.16	−0.07	−0.09	−0.11	−0.04	−0.08	**0.29**	**0.34**
SCL-90R Hos	0.07	**−0.25**	−0.08	−0.06	0.04	−0.12	−0.04	−0.02	−0.05	−0.11	0.01	**0.29**	0.19
SCL-90R Psy	0.03	−0.12	**−0.21**	−0.17	**0.21**	0.01	−0.02	−0.05	−0.15	−0.06	−0.11	**0.26**	**0.27**
SCL-90R Par	0.10	−0.12	**−0.22**	−0.14	0.11	−0.07	−0.06	−0.04	−0.15	−0.10	−0.07	**0.28**	**0.25**

Note. Sex was coded as 0 = male, 1 = female. Edu., education; RW, in remote working; HDI, house distress index; HR, Holmes-Rahe stress index; PSS, perceived stress scale; STAI, state-trait anxiety inventory; ECQ, existential concerns questionnaire; GHQ, general health questionnaire; Som, somatization; Hos, hostility/anger; Psy, psychoticism; Par, paranoia. Coefficients marked in boldface are significant at *p* < −0.1, and >0.20 or < −0.20.

**Table 5:** Semi-partial correlation coefficients for the three coping styles with psychological symptoms while controlling for all other psychological and socio-demographic factors.

	Engagement	Disengagement	Help-Seeking
PSS	**−0.17**	**0.27**	**0.32**
STAI	**−0.28**	**0.24**	**0.38**
ECQ Death anxiety	−0.06	**0.21**	**0.28**
GHQ	**−0.22**	**0.24**	**0.28**
SCL-90R Som	−0.07	**0.17**	**0.28**
SCL-90R Hos	−0.08	**0.16**	**0.19**
SCL-90R Psy	−0.10	**0.32**	**0.30**
SCL-90R Par	0.01	**0.17**	**0.17**

Note. PSS, perceived stress scale; STAI, state-trait anxiety inventory; ECQ, existential concerns questionnaire; GHQ, general health questionnaire; Som, somatization; Hos, hostility/anger; Psy, psychoticism; Par, paranoia. Coefficients marked in boldface are significant at *p* < 0.01.

## Data Availability

Publicly available datasets were analyzed in this study. This data can be found here: https://osf.io/8vec6/?view_only=65fd32ce214a40a6a546caa27cd9de94 (accessed on 1 November 2021).
